# Research progress and clinical translation prospects of the urinary tract microbiome in prostate cancer

**DOI:** 10.3389/fimmu.2026.1911969

**Published:** 2026-08-24

**Authors:** Ze-lin Li, Ruo-nan Qu, Shang-xuan Liu, Wei Wang

**Affiliations:** 1 Department of Urology, The Second Hospital & Clinical Medical School, Lanzhou University, Lanzhou, Gansu, China; 2 Department of Urology, Shenzhen Hospital, Southern Medical University, Shenzhen, China

**Keywords:** clinical translation, multi-omics, prostate cancer, tumor microenvironment, urinary tract microbiome

## Abstract

Prostate cancer (PCa) is one of the most common malignancies in men worldwide, and its development is influenced by multiple factors, including genetic susceptibility, hormonal dysregulation, chronic inflammation, immune dysregulation, and remodeling of the tumor microenvironment. In recent years, the urinary tract microbiome has emerged as an important component of the tumor ecosystem and has attracted increasing attention in PCa research. Accumulating evidence indicates that patients with PCa exhibit characteristic microbial alterations in urine, expressed prostatic secretions, semen, and prostate tissue, and that certain taxa are associated with tumor grade, stage, and recurrence risk. These microbes may participate in tumor initiation and progression through a variety of mechanisms, such as inducing chronic inflammation, activating signaling pathways including TLR/NF-κB and STAT3, modulating the Treg/Th17 balance, influencing macrophage polarization, and interfering with androgen metabolism. Meanwhile, advances in 16S rRNA sequencing, metagenomics, metatranscriptomics, and multi-omics integration have provided powerful tools for characterizing host–microbe interactions and their functional relevance. In addition, microbiome-based biomarkers derived from non-invasive samples such as urine, together with artificial intelligence and causal inference approaches applied to multi-cohort data, may offer promising opportunities for early screening, risk stratification, treatment monitoring, and personalized intervention in PCa. However, current evidence remains largely associative, and the causal relationship between microbial changes and PCa has not yet been fully established. Major challenges, including contamination in low-biomass samples and inter-cohort heterogeneity, continue to hinder clinical translation. Future research should focus on longitudinal cohort studies, multicenter validation, standardized sampling workflows, and mechanistic experiments to clarify key microbial signatures and their biological functions, thereby accelerating the clinical application of the urinary tract microbiome in precision diagnosis and treatment of PCa.

## 1 Introduction

Prostate cancer (PCa) is one of the most common malignancies among men worldwide and the fifth leading cause of cancer-related death. Complete epidemiological data for the past two years have not yet been finalized. Available data indicate that approximately 1.5 million new cases and 420, 000 deaths were reported globally in 2024. The incidence and mortality rates of PCa vary significantly across different regions. Generally, high-income countries exhibit higher incidence rates due to widespread screening, whereas economically disadvantaged regions, such as Africa and Latin America, experience higher mortality rates due to limited screening and treatment options (1, 2). Established high-risk factors include age, family history of cancer, genetic susceptibility, and Black race (3). In the United States, PCa has become the most common malignancy of the male reproductive system (4). Currently, the screening and diagnosis of PCa rely heavily on prostate-specific antigen (PSA) testing. However, PSA screening has significant limitations, including poor specificity, high false-positive rates, overdiagnosis, and the potential to trigger unnecessary invasive procedures and associated side effects (5–7). The U.S. Preventive Services Task Force (USPSTF) even recommends against routine screening for men aged 70 years and older (5). Therefore, there is an urgent need to identify novel and precise biomarkers to address this complex disease. In recent years, research has increasingly focused on improving the diagnostic capability for PCa through various novel biomarkers from urine and blood, as well as multiparametric magnetic resonance imaging (mpMRI), thereby optimizing screening pathways to reduce unnecessary interventions (8–10). Studies have shown that the development and progression of PCa are closely associated with the interaction with the tumor microenvironment (TME). The TME is a dynamic system composed of immune cells, vasculature, stromal cells, and the microbiota. Its aberrant regulation is inextricably linked to tumor progression and treatment response. As an integral component of the TME, the potential association between the microbiome and PCa has become a frontier research hotspot (11). Modern research has confirmed that the urinary tract, previously considered sterile, is in fact a microbial ecosystem rich in species and diverse in structure (12, 13). The urinary tract microbiota can not only mediate chronic inflammation and remodel the immune microenvironment through the induction of signaling pathways, but its distribution characteristics are also associated with PCa risk stratification (14). This paradigm shift has opened new avenues for investigating the urinary tract microbiome as a disease biomarker or therapeutic target (15, 16). Currently, there is limited literature focusing on the urinary tract microbiome in relation to PCa progression and clinical translation. This review systematically summarizes the frontier literature on the urinary tract microbiome and prostate cancer, aiming to elucidate the microbial characteristics of the urinary tract, key pro-carcinogenic mechanisms, causal evidence, and prospects for clinical translation, thereby contributing to the advancement of this field.

## 2 Research foundations and cognitive shifts of the urinary tract microbiome

This article is a narrative review aimed at synthesizing the literature on the relationship between the urinary tract microbiome and prostate cancer, and elucidating the underlying mechanisms. The discussion focuses on dysbiosis, oncogenic microbiota, inflammatory signaling crosstalk, epigenetic regulation, and clinical translational applications. The literature search was conducted using three major biomedical databases: PubMed, Web of Science, and Scopus. The search strategy combined two sets of terms: one set covered different subtypes of PCa, including localized PCa, advanced PCa, and biochemical recurrence; the other set focused on mechanisms related to the urinary tract microbiota, including keywords such as urinary tract microbiota, dysbiosis, inflammation-related cytokines, tumor microenvironment remodeling, and microbial biomarkers. This review prioritizes peer-reviewed English-language literature, with emphasis on recent cohort studies relevant to the scope of this review and preclinical basic research of significant mechanistic value. Additionally, reference lists of key original studies, meta-analyses, and similar reviews were manually screened to identify further relevant literature. Literature was prioritized for inclusion if it addressed the composition of PCa-associated urinary tract microbiota, predictive microbial signatures, or microbiota-modulating therapeutic strategies. Studies were excluded if they had weak relevance to the core theme of microbial mechanisms, lacked peer review, or had incomplete descriptions of sampling and sequencing methodologies. As this is a narrative review, rather than a standardized systematic review or meta-analysis, literature selection did not follow a pre-established quantitative integration framework. Instead, selection was based on thematic relevance and theoretical value for clinical translation.

## 3 Conceptual basis of the urinary tract microbiome

The urinary tract microbiome refers to the community of microorganisms colonizing or transiently residing in the urinary tract, along with their collective genome. Research subjects often include urine, prostatic fluid, semen, and prostate tissue. The presence of the urinary tract microbiome does not necessarily equate to urinary tract infection; rather, it is widely recognized as an important component in maintaining urogenital homeostasis (17).Therefore, greater attention should be paid to the interaction between this complex and dynamic system and host health and disease states. In the 1950s, Kass proposed the traditional concept of a “sterile urinary tract.” However, limited by outdated detection techniques, it was not until the end of the last century that advances in molecular biology technology completely overturned this long-held notion. Using methods such as bacterial culture, microscopy, and 16S rRNA sequencing, researchers confirmed the presence of microorganisms in urine. Furthermore, enhanced quantitative urine culture enabled the isolation of viable culturable bacteria, suggesting that the bladder is not a sterile environment (12). Over the past 15 years, technologies such as metagenomics and enhanced quantitative urine culture (EQUC) have further confirmed that the urinary tract microbiome can be present in asymptomatic healthy individuals (18). Subsequent studies have revealed that the urinary tract microbiome is closely associated with both urinary tract infections and urological tumors, with significant differences in microbial diversity and composition between healthy individuals and patients with urological cancers (12). This transition from “sterile” to “non-sterile” to “presence does not equal pathogenicity” to “inter-patient variability” not only marks technological progress but also suggests that the urinary tract microbiome may be closely involved in the progression of urological diseases.

The urinary tract microbiome can be derived from various tissue samples, each with corresponding focuses and limitations. Urine samples are the most commonly used and most suitable non-invasive sample source for large-scale clinical screening studies; however, they are highly susceptible to contamination, and using them alone makes it difficult to accurately reflect the true microbial composition (19). Prostatic fluid, although directly secreted by the prostate and better reflecting the local microenvironment, is difficult to collect, relies on operator technique, has a high failure rate, and is inevitably contaminated with urethral flora. Prostate tissue samples originate from the lesion and are theoretically the most suitable for mechanistic studies; however, confounding factors such as preoperative antibiotic use, spatial heterogeneity, and the risk of invasive contamination remain significant obstacles in research (19). In addition to the impact of different tissue sample sources, urinary tract microbiome samples are generally low-biomass samples, with a microbial load typically ranging from 10² to 10^6^ CFU/mL. They are highly susceptible to interference from factors including, but not limited to, age, history of urinary tract infections, metabolic status, sampling contamination, and sample storage contamination, leading to variability and instability in research results. Therefore, contamination control has become a critical determinant of study success (17, 20). In summary, the urinary tract microbiome not only aids in understanding the relevant microecological dysbiosis in PCa but also lays the foundation for subsequent analysis of its pathological features, immune mechanisms, and clinical translational value.

## 4 Urinary tract microbial characteristics in cancer samples

### 4.1 Urinary tract microbial characteristics associated with PCa development

The development and progression of PCa are closely linked to alterations in the urinary tract microbiota. Through analyses of samples including urine, prostatic fluid, and prostate tissue, multiple studies have revealed significant differences in microbial composition between PCa patients and healthy individuals or those with benign conditions. However, due to heterogeneity in sample sources, collection methods, sequencing technologies, and population backgrounds, study findings have been inconsistent. **Table 1** systematically summarizes the key findings from different sample types.

**Table 1:** Summary of studies on cancer samples and urinary microbiota in different research PCa-Prostate cancer, BPH-Benign prostatic hyperplasia.

Sample type	Study subjects/group	Key finding	Key microbiota	References
Urine	PCa patients vs benign biopsy patients	The urinary tract microbiome of PCa patients is significantly separated from controls and correlates with Gleason scores	Enrichment of Veillonella and Streptococcus, reduction of Faecalibacterium.	(21)
Urine	PCa patients	Increased abundance of specific bacteria in PCa patients	Anaerococcus, Dialister, etc.	(22)
Urine	PCa patients vs BPH patients	Significant remodeling of urinary tract microbiome diversity and community structure in PCa patients	Enrichment of Bacteroidetes, α-Proteobacteria, etc.; reduction of Actinobacteria and Dehalobacterium	(23, 24)
Urine	High-grade/Intermediate-high risk PCa vs Low-grade/Low risk patients	Higher abundance of certain microbial communities in urine of high-grade PCa and intermediate to high-risk late-stage patients	Unclassified Streptococcus, Ureaplasma, Mycoplasma, etc.	(22)
Expressed Prostatic Secretion	PCa patients vs Non-PCa patients	Increased abundance of specific microbial communities in tissues of PCa patients	Enterobacter, Lactobacillus, Streptococcus, etc. .	(25)
Expressed Prostatic Secretion/Semen	PCa patients vs Non-PCa patients	Decreased microbial diversity in the PCa patient group, with increased abundance of certain genera	Alkaliphilus, Enterobacter, Lactobacillus, etc.	(26)
Prostate Tissue	PCa tissue vs Benign/Adjacent tissue	Increased abundance of specific genera in tissues of PCa patients, potentially involved in PCa progression	Propionibacterium, Staphylococcus, Sharpea, etc.	(27–29)
Prostate Tissue	PCa tissue vs Benign tissue	Specific genera enriched in PCa or normal tissues	Staphylococcus is enriched in PCa, while Streptococcus is enriched in benign tissues.	(30, 31)
Prostate Tissue	High-grade PCa vs Low-grade PCa	Specific genera enriched in high-grade PCa, potentially promoting tumor proliferation	Bacillus, pro-inflammatory Mycobacterium, etc.	(32–34)
Prostate Tissue	Advanced stage PCa vs Localized stage	Enrichment differences of specific bacteria in different stages of PCa	Lactobacillus enriched in advanced stages, Ensifer enriched in localized stages.	(35)
Prostate Tissue	PCa and Cutibacterium acnes	This bacterium can promote PCa cell proliferation and is significantly associated with inflammation	Propionibacterium acnes of the prostate adaptive subtype.	(36, 37)
Prostate Tissue	High-Gleason-score PCa tissues	Higher abundance of partial bacterial flora	Corynebacterium, Mycobacterium, Cutibacterium, Pelomonas	(38)

### 4.2 Urinary tract microbial characteristics associated with PCa progression and prognosis

Based on figures and recent studies, the urinary tract microbiome of PCa patients differs from that of healthy individuals or patients with benign diseases in terms of composition and abundance. Overall, these differences manifest as a decrease in microbial diversity, an increase in certain pro-inflammatory and anaerobic bacteria, and the association of certain microbial taxa with higher-risk stratification, higher grade, and poorer prognosis. These findings provide preliminary evidence for a link between the urinary tract microbiome and PCa. From the perspective of PCa development, compared with benign patients or healthy individuals, PCa patients exhibit alterations in specific genera, such as decreases in Escherichia and Enterococcus, and increases in Veillonella, Streptococcus, and Bacteroides. The dominant taxa in these differences may be associated with chronic inflammation and oxidative stress. However, as most of these findings are derived from cross-sectional studies, it remains difficult to determine whether the microbial changes are a cause or a consequence of PCa development, or whether both are manifestations of other confounding factors. Regarding disease progression, some studies suggest that urinary tract microbial characteristics may be associated with PCa stage and grade. For example, Corynebacterium and Mycobacterium show higher abundance in tissues with high Gleason scores (38); Corynebacterium is more abundant in high-grade PCa; and the detection of anaerobic bacteria such as Fusobacterium is associated with an increased risk of biochemical recurrence and metastasis (22). However, given the generally small sample sizes of existing studies, these results require further validation in larger, standardized cohorts.

### 4.3 Reasons for heterogeneity in study findings

Although existing studies have accumulated some evidence regarding PCa-related microbial alterations, and most have identified certain common taxa, considerable contradictions remain across different research groups and cohorts. These include completely opposite conclusions regarding dominant taxa, inconsistent associations between specific genera and PCa, and divergent patterns of microbial diversity (32, 34, 36, 37, 39, 40). These discrepancies may primarily arise from the following factors. First, differences in sample source may be one of the most significant contributors to result variability (41). One study simultaneously collected urine, urethral swabs, and prostate biopsy tissue from the same cohort and performed sequencing analysis, finding that the urinary microbiota closely resembled the glans penis microbiota, yet both were significantly different from the prostate tissue microbiota, with substantial intergroup dissimilarities (40). Additionally, in another study by Okada analyzing prostate tissue and urine samples from the same patients, significant differences were observed in both diversity indices and specific microbial taxa between the two sample types (42). Furthermore, different urine collection methods and different approaches for obtaining prostate tissue can also significantly affect the detection results from the same patient. Collectively, these findings suggest that different tissue samples may reflect distinct microbial ecological niches at different anatomical sites, and when combined with issues of contamination, the results may not fully represent the true *in situ* environment, leading to bias. Moreover, the focus and advantages of different sample types are not identical, and they cannot be simply compared across studies in a straightforward manner. Similarly, with advances in sequencing technology, 16S amplicon sequencing and metagenomic sequencing have been increasingly applied in urinary tract microbiome research. However, the technical challenges and limitations inherent to these two approaches may also contribute to the heterogeneity of study findings. 16S amplicon sequencing is low-cost and widely used, but it typically provides taxonomic information only at the phylum-to-genus level, has limited resolution for distinguishing closely related species, and is prone to missing low-abundance microorganisms that are poorly represented in reference databases, potentially leading to divergent results (43). In contrast, metagenomics enables identification at the species to strain level and allows comprehensive analysis of functional genes and pathways; however, its high cost and susceptibility to host DNA contamination are critical drawbacks, especially for low-biomass samples such as those from the urinary tract (44, 45). Second, racial and geographic differences can lead to inherent biological variation among populations, representing another source of result heterogeneity (29). One study compared the prostate microbiota of PCa patients from different racial and geographic backgrounds and found that, compared with non-African samples, African samples exhibited increased bacterial abundance and richness. Moreover, genera such as Bacteroides, Firmicutes, Prevotella, and Fusobacterium were detectable only in African samples, and these characteristics may be associated with higher tumor mutational burden (46). Although this phenomenon may, to some extent, reflect genuine biological differences driven by environmental factors, the small sample size of this study and the lack of similar studies mean that statistical error and contamination effects cannot be ruled out. Finally, the most easily overlooked factor contributing to result heterogeneity is contamination. Many previously reported taxa considered characteristic of PCa have actually been identified as contaminants introduced during sampling or laboratory procedures. Salachan et al. found that high-abundance genera in prostate tissue, such as Acinetobacter, Enterobacter, and Escherichia, are common sequencing contaminants. In another study of 65 patients who underwent prostatectomy, the most abundant genera identified, including Pseudomonas and Cutibacterium, showed no difference compared with adjacent benign tissue and were similarly considered to be contaminants introduced during the experiment (33, 47). These contaminants may originate from sampling sites such as skin, rectum, or urethra, or from laboratory equipment and instruments, and false-positive results may even be introduced during sequencing analysis. It is evident that rigorous contamination control strategies are crucial for obtaining accurate and reliable results.

## 5 Potential biological mechanisms

### 5.1 Microbial dysbiosis and chronic inflammation

**Figure 1:**
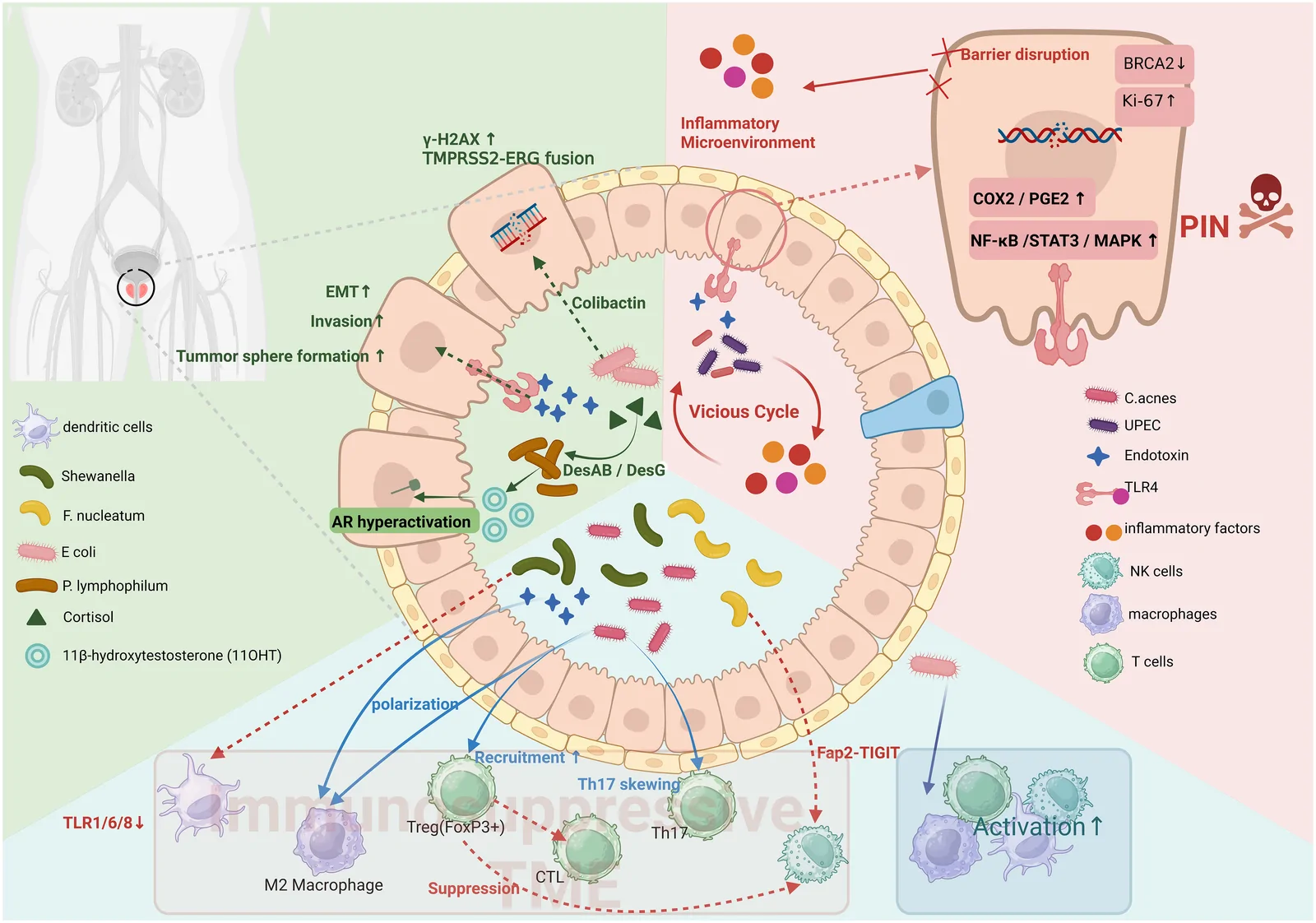
Microbial mechanisms linking the urinary tract microbiome to prostate cancer.

Chronic inflammation is a critical driver of PCa development and progression. Multiple epidemiological studies have suggested a positive association between prostatic inflammation and the risk of PCa (48). Chronic inflammation can promote tumor progression through various mechanisms, including enhanced cell proliferation, release of inflammatory cytokines, and DNA damage (25, 31). Persistent microbial infection may contribute to the establishment and maintenance of chronic prostatic inflammation (49). Concurrently, the inflammatory microenvironment induced by various damaging factors can disrupt local microbial homeostasis, ultimately leading to a vicious cycle in which chronic inflammation and dysbiosis mutually reinforce and exacerbate each other (50). As illustrated in **Figure 1**. Based on a database study from Taiwan, Pan et al. further observed a statistical association between prostate cancer and urinary tract infection; however, this study did not support a direct causal role of urinary tract infection in prostate cancer (51). Therefore, although urinary tract infection and related microbial dysbiosis cannot be definitively established as independent carcinogenic factors, existing evidence suggests that microorganisms may constitute part of the inflammatory background of PCa.

Cutibacterium acnes (formerly Propionibacterium acnes) is a Gram-positive bacillus that has been frequently mentioned in this field in recent years, with multiple experimental studies suggesting its potential role in PCa development and progression (28). C. acnes has been demonstrated to possess hemolytic, cytotoxic, and immunostimulatory activities. It may stimulate prostate epithelial cells to secrete various chemokines, recruit peripheral immune cell infiltration, and ultimately establish a pro-inflammatory microenvironment characterized by cytokines such as TNF-α, VEGF, IL-1β, IL-6, IL-8, IL-12, and IL-17 (52, 53). In a co-culture experiment, infection of benign prostatic PNT1A cells with C. acnes led to increased proliferation rates in the short term, accompanied by enhanced secretion of certain chemokines. Similarly, RWPE1 cells infected with C. acnes actively secreted IL-6, IL-8, and other chemokines, and further activated the COX2-prostaglandin, NF-κB, STAT3, and plasminogen-matrix metalloproteinase pathways (53). Another *in vitro* study using prostate epithelial cells showed that C. acnes could enter infected cells, localize within perinuclear vesicles, impair homologous recombination repair, and downregulate BRCA2 levels, suggesting that it may induce a “BRCAness” phenotype and increase genomic instability and cancer risk (54, 55). The basis for the effects of C. acnes on the prostate may be related to its ability to persist within prostate epithelial cells (56). Animal experiments have shown that inoculation of C. acnes isolated from human prostatectomy specimens into C57BL/6J mice induced severe inflammatory changes in the dorsal lobe of the mouse prostate. The formation of inflammatory foci and the increase in Ki-67 proliferation index were dependent on viable bacterial invasion, and immunohistochemistry and *in situ* hybridization further confirmed the persistent presence of this bacterium within prostate epithelial cells (56).

Uropathogenic Escherichia coli (UPEC) and its intrinsic cell wall component lipopolysaccharide (LPS) have been demonstrated to promote PCa progression by inducing chronic inflammation. Repeated colonization of the prostate by UPEC may lead to chronic prostatitis, which subsequently results in prostatic intraepithelial neoplasia and high-grade atypical hyperplasia, accompanied by DNA damage accumulation and the formation of precancerous lesions (57). In various prostate cell lines, LPS can bind to the TLR4/MD-2 complex and activate the MyD88-dependent pathway, upregulating inflammatory mediators such as IL-6, IL-8, COX2, and TNF-α, and further triggering the activation of NF-κB, STAT3, Wnt, and p38MAPK pathways, while simultaneously inhibiting autophagy and increasing CCL2 expression, thereby maintaining an inflammatory state (38). In addition, animal experiments have confirmed that UPEC infection can induce fibrocyte recruitment and promote collagen deposition through a CCR2-dependent pathway, mediating fibrotic remodeling of prostate tissue. This suggests that E. coli not only triggers local inflammation but also drives the progression of prostatic stromal fibrosis (58). Another 12-week chronic infection model in mice further confirmed that persistent UPEC stimulation induces chronic, persistent prostatic inflammation. Over the course of infection, prostate tissue gradually developed precancerous lesions such as prostatic intraepithelial neoplasia and high-grade epithelial dysplasia. Concurrently, the proliferation activity of prostate epithelial cells was upregulated, oxidative DNA damage was exacerbated, and the expression levels of androgen receptor and the tumor suppressor gene PTEN were downregulated (57). Existing reviews have consistently concluded that the E. coli-mediated chronic inflammatory microenvironment can disrupt the integrity of the prostate epithelial barrier, recruit various inflammatory cell infiltrates, and release large amounts of reactive oxygen species, ultimately driving the development and progression of PCa precursor lesions, including proliferative inflammatory atrophy and prostatic intraepithelial neoplasia (14).

### 5.2 Remodeling of the immune microenvironment

Urinary tract microorganisms can further remodel the local immune microenvironment on the basis of inducing chronic inflammation, and their effects are species-dependent. Studies have demonstrated that persistent stimulation by certain bacteria may influence the infiltration and effector functions of local immune cells, tending to establish an immunosuppressive microenvironment that promotes tumor immune evasion. Conversely, some commensal bacteria and their metabolites may reshape the immune microenvironment and enhance the sensitivity of PCa to immunotherapy by promoting CD8^+^ T cell activation and enhancing antigen presentation.

Cutibacterium acnes has been considered to possess strong carcinogenic relevance in previous experiments. *In vitro* studies have shown that C. acnes infection can induce the polarization of human macrophages toward the M2 phenotype, upregulate immunosuppression-related molecules such as CCL17 and PD-1, and promote the recruitment and infiltration of Treg cells. In addition, the elevated IL-6 level in the inflammatory microenvironment formed by C. acnes favors the establishment of the Th17 lineage, and Th17-secreted cytokines such as IL-17A and IL-22 can enhance the viability, migration, and invasion of PCa cells. Under the influence of C. acnes, the Treg/Th17 balance is disrupted, and the anti-tumor immune response can be weakened by inhibiting the functions of effector T cells, NK cells, and antigen-presenting cells, as well as by regulating the activities of myeloid cells and T cells (14, 59, 60). Studies have further suggested that reducing the C. acnes load or blocking downstream inflammatory axes may help restore the Treg/Th17 balance and remodel the immune microenvironment, representing a potentially intervenable target in future therapies (61, 62). As mentioned above, the Escherichia coli component LPS can activate the NF-κB and MAPK pathways in various PCa cell lines, such as PC3 and DU145, upregulate inflammatory mediators, promote epithelial-mesenchymal transition, enhance the invasion and metastatic capacity of PCa, and facilitate tumor immune evasion (38). However, the effects of LPS from different sources should not be generalized; LPS from different origins can trigger distinct degrees of innate immune signaling due to differences in their chemical structures. For example, LPS from Porphyromonas gingivalis has been experimentally shown to fail to induce PD-L1 expression in DU145 cells, suggesting that it may not be involved in tumor immune evasion in this model (63). In addition, LPS concentration, mode of administration, and duration of exposure also influence its effects. A single short-term LPS stimulation may only induce acute inflammation, during which the body can still maintain homeostasis, whereas long-term repeated exposure is more likely to drive the formation of an immunosuppressive microenvironment (38, 64, 65). Another clinical cohort study involving 830 PCa patients found that enrichment of Helicobacter pylori was accompanied by macrophage M2 polarization, upregulation of IL-17RA and cathepsin K, followed by activation of epithelial-mesenchymal transition, increased risk of metastasis, and association with poorer prognosis (66).

TLRs are key receptors of the innate immune system for recognizing pathogen-associated molecular patterns. Attenuation of TLR signaling indicates a reduced capacity of the host to sense and eliminate microorganisms. A whole-transcriptome sequencing study found that in PCa tissues with high abundance of Shewanella, the expression of immune-related genes such as TLR1, TLR6, and TLR8 was downregulated, accompanied by a decreased enrichment score for dendritic cells, further suggesting impaired innate immune recognition and antigen presentation capacity (33, 52). Notably, unlike most microbiota, Shewanella may not drive tumorigenesis through inflammation, as most malignant tissues in that study showed no significant histological inflammation. Its role in PCa development may be more related to a vicious cycle between reduced host innate immune recognition and the persistent survival of Shewanella. However, this hypothesis requires further confirmation through causal and correlational studies (33). In addition, Fusobacterium nucleatum can bind to the inhibitory receptor TIGIT on the surface of NK cells through its virulence factor Fap2, thereby inhibiting the cytotoxic activity of NK cells against tumor cells and weakening innate immune surveillance (67, 68). This type of mechanism, which directly acts on immune cells, further expands the repertoire of pathways through which urinary tract microorganisms mediate immune evasion. Some studies have also indicated that Fusobacterium nucleatum and Shewanella may downregulate immune genes such as TLR3 and TGFB2, thereby inhibiting immune cell function and promoting tumor immune evasion (31). However, research in this area remains limited and requires further validation.

Conversely, certain bacteria may play a role in improving the immune microenvironment. A pathogenic Escherichia coli strain (CP1) can colonize the MYC/PTEN model. When combined with PD-1 inhibitors, CP1 increased the infiltration of mature dendritic cells, CD8^+^ T cells, Th17 cells, M1 macrophages, and NK cells within tumors, suggesting simultaneous activation of both innate and adaptive immunity. At the same time, it reduced the number of Treg cells and VEGF expression, thereby lowering the level of immunosuppression and improving the angiogenic environment, which favors sustained anti-tumor immunity (38, 69). Therefore, CP1 may contribute to the conversion of “immunologically cold” tumors to “immunologically hot” tumors by activating multiple anti-tumor immune cell types, potentially helping to overcome resistance to PD-1 blockade (38).

### 5.3 Direct regulation of PCa by microbiota-derived active molecules

Active molecules derived from urinary tract microorganisms refer to substances produced or released by urogenital and prostate-associated microbiota during colonization, metabolism, or infection that can influence host cell functions. These molecules primarily include microbial metabolites, toxins, and other pathogenicity-associated molecules, and they may play roles in inducing DNA damage, enhancing the metastatic capacity of cancer cells, and promoting PCa proliferation.

Multiple studies have confirmed that colibactin secreted by Escherichia coli strains carrying the pks gene cluster can efficiently induce genomic DNA double-strand breaks (DSBs) in prostate cancer cells. An *in vitro* study published in Proceedings of the National Academy of Sciences (PNAS) demonstrated that transient infection of LNCaP cells with pks^+^ E. coli resulted in a significant increase in the comet tail moment and elevated expression of γ-H2AX, a specific marker of DSBs. The extent of DNA damage induced by this infection even exceeded that caused by 8 Gy of ionizing radiation. Furthermore, fluorescence *in situ* hybridization (FISH) results confirmed that this damage directly led to the separation of TMPRSS2 and ERG gene loci signals, resulting in chromosomal rearrangement and the generation of TMPRSS2-ERG gene fusions (70). Androgen signaling can further synergistically amplify this carcinogenic effect. A clinical cohort study involving 235 individuals confirmed that colonization with pks^+^ E. coli was significantly associated with the incidence of PCa. *In vitro* cell experiments also found that co-treatment with colibactin and dihydrotestosterone exacerbated replication fork stalling, significantly increased the frequency of chromosomal fusion events, and induced a large number of somatic mutations and kataegis mutational clusters, with the synergistic effects of the two agents continuously disrupting the genomic stability of prostate cells (71). In addition, relevant reviews have clearly indicated that colibactin drives genomic instability by inducing DNA double-strand breaks and DNA interstrand crosslinks, and may synergize with androgens to stimulate chromosomal translocations and promote the generation of oncogenic fusion genes such as TMPRSS2:ERG (72).

Lipopolysaccharide (LPS), a cell wall component of Gram-negative bacteria, acts as a core inflammatory stimulus in the urinary microenvironment and can significantly drive the progression of malignant phenotypes in advanced PCa. Studies have found that LPS treatment generally enhances the migration and invasion abilities of multiple PCa cell lines, while also increasing colony formation and three-dimensional tumor sphere formation capacity, indicating that the potential for distant metastasis and cancer stem cell properties are significantly activated (38). Another *in vitro* study further demonstrated that LPS can act on various PCa cells, including DU145 and PC3, to promote cell invasion and migration and induce tumor sphere formation. Moreover, LPS effectively enriched the CD44^+^CD133^+^ cancer stem cell subpopulation and upregulated the expression of classical stemness markers such as SOX2 and Nestin, thereby maintaining an undifferentiated malignant phenotype (73). At the mechanistic level, this study confirmed that LPS-mediated remodeling of tumor stemness is highly dependent on STAT3 signaling activation. Inhibition of STAT3 phosphorylation could significantly downregulate the secretion of the pro-inflammatory cytokines IL-6 and IL-8, while simultaneously reversing the elevation of stemness markers and the capacity for tumor sphere formation (73). In addition, it has been established that LPS initiates downstream NF-κB, STAT3, Wnt, and p38 MAPK signaling pathways through the TLR4 receptor. These pathways collectively regulate PCa cell survival, proliferation, epithelial-mesenchymal transition (EMT), and the accumulation of stem cell properties. LPS is therefore considered one of the key inflammatory mediators through which urinary microbiota dysbiosis drives the progression of advanced PCa (38).

Microorganisms in the urogenital tract can also generate functional androgen molecules through their unique steroid metabolic pathways, thereby continuously activating the androgen receptor and driving the proliferation of androgen-dependent PCa cells. This represents an exogenous trigger for aberrant local AR signaling activation. The urinary tract strain Propionimicrobium lymphophilum can catalyze the conversion of host cortisol into 11β-hydroxytestosterone via the key enzymes DesAB and DesG. In a co-culture system, this derivative significantly promoted the proliferation of LNCaP cells (74). The study also detected the metabolic activity of converting cortisol into 11OHAD and 11OHT in some human urine samples, and successfully isolated the corresponding steroidogenic bacterial strains, directly suggesting that the *in situ* urinary tract microbiota can accomplish the local conversion of androgen precursors into active androgens (74).

## 6 Evidence of association and causality between the urinary tract microbiome and prostate cancer

Recent research findings indicate a stable association between the urinary tract microbiome and PCa. A review encompassing nine studies and a total of 611 PCa patients found that, overall, there was no significant difference in the α-diversity of urinary bacteria between PCa patients and controls. However, certain genera, including Streptococcus, Bacteroidetes, and Faecalibacterium, were relatively more abundant in PCa patients. Additionally, the presence of unassigned Streptococcus and Alloscardovia omnicolens was associated with higher-grade PCa (39). This suggests that alterations in the urinary tract microbiome are more likely to reflect a structural remodeling of the microbial community rather than a simple change in diversity (39). Studies using other sample types, such as prostate tissue, prostatic fluid, and post-prostatic massage urine, have further corroborated this trend. Previous research has detected Cutibacterium acnes in prostate specimens, and its presence was more common in the PCa group; however, no statistically significant association was found with subsequent PCa development, inflammation, or Gleason score (39). In expressed prostatic secretion samples, reduced microbial diversity was observed in PCa patients, accompanied by enrichment of genera such as Streptococcus, Bacillus, Enterobacteriaceae, and Lactococcus (28). In post-PM urine samples, PCa patients exhibited elevated abundances of Veillonella, Streptococcus, and Bacteroides, while the abundances of Faecalibacterium, Lactobacillus, and Acinetobacter were decreased (39). Collectively, these results suggest that consistent microbiota differences exist across various types of urogenital samples, and these differences may be associated with PCa status. Other studies have found that specific anaerobic bacteria may be linked to disease risk stratification. A PCa cohort study showed that when any of the following ABBS anaerobic genera—Anaerococcus, Bulleidia, BVAB3, or Sneathia—was detected in urine sediment, the proportion of clinically more aggressive cancers was higher. Similarly, the detection of five anaerobic genera in urine sediment, urinary extracellular vesicles, and tumor tissue was associated with an increased risk of disease progression (39). Furthermore, Cutibacterium acnes and Cutibacterium granulosum have been repeatedly reported to be enriched in the urine of PCa patients (17). Another notable species is Propionimicrobium lymphophilum, which can not only be detected in urine but is also considered to possess androgen-synthesis-related capabilities, potentially linking it to PCa development (38). Ureaplasma urealyticum and Ureaplasma parvum have also been frequently reported to be associated with PCa and higher-grade tumors (39). Overall, these findings indicate that specific members of the urinary tract microbiome may be associated with the occurrence, progression, and risk stratification of PCa.

Although the aforementioned studies suggest a relatively stable association between the urinary tract microbiome and PCa, evidence for a causal relationship between the two remains limited at this stage. Most existing studies are cross-sectional or case-control in design and lack longitudinal follow-up. While they primarily reveal the existence of microbial differences, it is difficult to determine whether these changes are a cause preceding the disease or a consequence of local microenvironment remodeling following tumor formation (39, 75). Various confounding factors, such as prostate volume, lifestyle, lower urinary tract symptoms, and other host factors, may also alter the characteristics of the urinary tract microbiome, thereby interfering with its true association with PCa (75). At the same time, research on the urinary tract microbiome faces significant sampling and technical biases. Microbial signals in urine may originate from the bladder, urethra, or even external skin or genital sites, and may not accurately reflect the local microbial ecology of the prostate. Furthermore, inconsistent results across different sample types, including urine, EPS, semen, and tissue samples, further complicate causal interpretation (28). Current research on PCa and the urinary tract microbiome predominantly supports the existence of an association. However, due to limitations in study design, sample source, and control of confounding factors, high-quality evidence sufficient to confirm a causal relationship is still lacking. Future studies combining prospective longitudinal designs, standardized sampling strategies, and mechanistic validation experiments are needed to further elucidate the true role of the urinary tract microbiome in PCa and its potential clinical applications.

## 7 Application of multi-omics integrative studies in the urinary tract microbiome and PCa

### 7.1 Application of microbiome and host immunome

Multi-omics integrative research refers to the combined collection and comprehensive analysis of data from multiple molecular levels, including the immunome, genomics, transcriptomics, the microbiome, and metabolomics. This approach provides a more comprehensive biological understanding than any single omics platform alone, thereby enabling a systematic dissection of the complex regulatory mechanisms and network interactions in organisms under both healthy and diseased states (76, 77). In recent years, multi-omics integrative studies have significantly improved the accuracy of disease risk prediction, early diagnosis, prognosis, and treatment response, and have become a core research tool in precision oncology. Previous literature on the urinary tract microbiome has often focused on single-omics analyses of microbial data alone. While such data are intuitive and yield clear results, they are insufficient to fully elucidate how the microbiota influences the host, whether it acts through specific pathways, and whether it is genuinely involved in the occurrence of PCa. With recent technological advances, multi-omics research has made initial progress in this field, with the integration of “microbiome and host transcriptome” being one of the most mature directions. Salachan et al. performed microbial annotation through total RNA sequencing of prostate tissue, comparing microbial differences between benign and malignant tissues, as well as among samples with different aggressiveness grades and biochemical recurrence status. Concurrently, they conducted host differential gene expression and cell-type enrichment analyses. They found that Shewanella was enriched in malignant tissues and was associated with downregulation of TLR signaling and a reduction in dendritic cells, suggesting that microorganisms may remodel the local immune environment. In addition, they observed that malignant tissues were enriched in M1/M2 macrophages compared to benign tissues, which may further reflect the disruptive effects of microorganisms (33). Another study showed that low-biomass microbial DNA in prostate tissue could be repeatedly detected, and that multiple bacterial and viral signals could converge on tumor-related pathways such as TLR-NF-κB, MAPK, PI3K/AKT/mTOR, cGAS-STING, and p53/pRb, suggesting that microbial genes and metabolites may be involved in chronic inflammation, oxidative stress, and other processes (78).

### 7.2 Application of microbiome and metabolome

Musleh et al. performed 16S rRNA sequencing and gas chromatography-mass spectrometry analysis of volatile metabolites in urine samples from a radical prostatectomy cohort. They found that PCa patients exhibited higher urinary microbial diversity and enrichment of certain genera compared to BPH patients. Among 36 volatile organic metabolites identified in urine, 14 showed significant differences between the two groups. Furthermore, PCa-associated genera were positively correlated with these metabolites, suggesting that the synergistic alterations of the microbiota and metabolites may serve as potential non-invasive diagnostic clues in the future (24). Compared to the urinary tract microbiome, the field of the gastrointestinal microbiome appears to be more mature. Tan et al. reviewed that short-chain fatty acids, secondary bile acids, and polyamines produced by the gut microbiota can influence the prostatic inflammation threshold, barrier integrity, and immune response through systemic circulation. These metabolites, detectable by liquid chromatography-mass spectrometry or GC-MS, may have the potential to be incorporated into laboratory biomarker panels (79). Notably, Liu employed a two-sample Mendelian randomization approach and found that 11 gut microbial taxa had a causal association with PCa. They further revealed the mediating roles of sex hormones and blood metabolites in this relationship, providing integrative evidence at the causal level for the “gut microbiota–metabolism–PCa” axis (80).

### 7.3 Intra-microbiome multi-omics integration

The urinary tract microbiota represents a low-biomass sample type, and sample contamination has long been a recognized issue. Furthermore, the commonly used 16S rRNA sequencing approach yields suboptimal analytical results for low-biomass samples, as it can only reflect the presence or absence of microorganisms without reaching the level of functional and pathway analysis. Consequently, recent years have seen an increasing emphasis on the use of higher-resolution methods to determine the functional activity of the microbiota. Feng et al. proposed that metagenomic sequencing can resolve the species composition and the landscape of potential functional genes within the microbial community, but it cannot distinguish whether the detected nucleic acids originate from living microorganisms or from residual DNA released by dead microbes. In contrast, metatranscriptomic sequencing can directly characterize the real-time transcriptional activity of microbial genes. The combined application of these two techniques enables, to a certain extent, the precise screening of functional microorganisms involved in host-microbe interactions, with enhanced reliability (28, 31). Similarly, Salachan et al. achieved metatranscriptomic-level resolution of the microbiome by annotating microbial sequences from total RNA sequencing data. This approach moves beyond the limitations of simple species composition analysis and shifts the focus toward the study of functionally active microbial communities, thereby facilitating a better understanding of the interaction mechanisms between microorganisms and the prostatic epithelium within the urinary microenvironment (33). These research strategies indicate that moving beyond descriptive studies of species composition alone may effectively reduce interference from confounding signals and provide a robust methodological framework for uncovering molecular mechanisms and resolving controversies in this field.

### 7.4 Multi-cohort integration and artificial intelligence-driven data modeling

With the continuous accumulation of multi-omics datasets, related research is transitioning from the descriptive characterization of single cohorts toward cross-cohort validation, reproducibility analysis, and the construction of prognostic and predictive models. At the level of multi-cohort integration combined with artificial intelligence modeling, Zhou et al. proposed that raw multi-cohort, multi-omics data could be standardized and incorporated into a unified database. By leveraging deep learning and generative AI, microbial features can be mined, and complex host-microbe interactions can be dissected, thereby establishing associations between microbial signatures and the clinical outcomes of prostate cancer (81). From the perspective of multi-omics network analysis, Wang et al. argued that integrating microbiome, genomics, transcriptomics, proteomics, and metabolomics data, and constructing multi-level regulatory networks through cross-omics association analysis, could help elucidate underlying regulatory mechanisms and identify novel diagnostic biomarkers and potential therapeutic targets (31). Concurrently, advancements in statistical methods are further empowering mechanistic interpretation. Liu et al. combined Mendelian randomization with mediation analysis, enabling both the screening of key microbial taxa with causal effects and the quantitative assessment of the mediating roles of sex hormones and circulating metabolites in the relationship between the microbiota and tumor phenotypes. This exemplifies a frontier research paradigm that integrates statistical causal inference with the dissection of biological mechanisms (80). The complementary nature of data integration, AI, and causal statistical frameworks may help mitigate the current challenges of high cohort heterogeneity and inconsistent results in urinary tract microbiome research. These approaches may facilitate the systematic elucidation of the complete regulatory pathways through which the microbiota participates in the initiation and progression of PCa. However, the development and application of these approaches in this field still require extensive external validation.

## 8 Translational medicine background and clinical strategies

### 8.1 Microbial biomarkers: from candidate signals to clinical validation

The urinary tract microbiota and its associated molecular components hold promise as candidate biomarkers for PCa screening, risk stratification, prognostic assessment, and treatment response monitoring. However, their clinical value is currently best positioned as a complement to PSA, mpMRI, pathological grading, and clinical staging. Candidate signals are primarily derived from urine, post-prostatic massage urine (post-PM urine), prostatic fluid, semen, and prostate tissue. Among these, urine collection is convenient, making it suitable for non-invasive screening and dynamic monitoring, while tissue samples are more closely related to the lesion and are therefore more appropriate for mechanistic studies and localized risk stratification. As urogenital samples are generally low-biomass systems, biomarker studies should simultaneously report sampling methods, antibiotic exposure, negative/positive controls, DNA extraction batches, sequencing platforms, decontamination procedures, and statistical models. Studies should also adhere to the low-biomass microbiome reporting standards and the STORMS (Strengthening The Organization and Reporting of Microbiome Studies) guidelines to enhance transparency and reproducibility (82, 83).

From the perspective of diagnosis and risk stratification, urinary tract microbiome classification models, urinary volatile metabolite profiles, and urinary nuclear magnetic resonance (NMR) metabolomics analyses have all demonstrated exploratory value in distinguishing PCa from benign disease (24, 84, 85). The anaerobic bacterial biomarker panel proposed by Hurst et al. was found to be associated with the risk of disease progression in samples such as urine sediment and tumor tissue, suggesting its potential as a prognostic indicator for biochemical recurrence, thereby identifying patients with more aggressive disease or a higher risk of recurrence (22, 25). Furthermore, certain genera in prostate tissue may interact with PSA levels, and combined detection could potentially assist in improving the diagnostic accuracy for patients in the PSA gray zone. Genera such as Fusobacterium, Shewanella, Enhydrobacter, and Lautropia exhibit differential abundance in tumor tissue, and this differential distribution may suggest microbial involvement in the regulation of tumor aggressiveness, potentially correlating with risk stratification or prognosis (34, 35, 52). However, these results support the role of microbial features as incremental variables in multimodal predictive models, and they are currently insufficient to independently guide decisions regarding biopsy or treatment escalation.

The current major bottlenecks in microbial biomarker research include limited sample sizes, predominantly cross-sectional study designs, inconsistencies in sampling and sequencing workflows, insufficient control of confounding factors, and a lack of external validation. Microbiota differences across different sample sources cannot be simply substituted for one another. Furthermore, microorganisms detected in tumor tissue may be influenced by preoperative antibiotics, tissue processing, host DNA background, and batch effects. Therefore, before a candidate biomarker can transition from a statistical association to a clinical application, it must at least undergo fixed algorithm training, external validation, multicenter prospective cohort validation, calibration curve analysis, and decision curve analysis. It is also essential to clarify whether these biomarkers truly add diagnostic and therapeutic value beyond PSA density, PI-RADS scores, ISUP grading, and clinical staging (86).

A more feasible translational pathway for the future is the integration of the microbiome, metabolome, and host immunity. Using urine or post-PM urine as a non-invasive entry point, combined with metagenomics or metatranscriptomics to improve species and functional resolution, and further integrating metabolites, inflammatory factors, T cell/myeloid cell infiltration markers, and imaging data, an interpretable risk model can be established. Such models should predefine clinical endpoints, such as positive biopsy rates, detection rates of clinically significant PCa, biochemical recurrence, and response to radiotherapy or immunotherapy. Only through repeated validation across different populations, regions, and experimental platforms can these models truly enter the realm of precision diagnosis and treatment (84).

### 8.2 Therapeutic targets of microbial intervention

At the therapeutic level, modulating the urinary tract microbiome provides a conceptual foundation for adjuvant therapy in PCa. However, current evidence supports the notion of “intervenable targets” rather than mature clinical protocols. Potential strategies include supplementing with probiotics or prebiotics, targeted elimination of pro-inflammatory or pro-carcinogenic bacteria, utilizing commensal or engineered bacteria to activate anti-tumor immunity, blocking harmful microbial metabolites, modulating immune-metabolic signals such as short-chain fatty acids and bile acids, and improving responses to endocrine therapy, radiotherapy, or immunotherapy through systemic microbial ecological interventions (87, 88). The common goal of these strategies is to reduce chronic inflammation and immunosuppression, enhance antigen presentation as well as the functions of CD8+ T cells and NK cells, and attenuate the activation of metabolic or signaling pathways associated with treatment resistance.

For probiotics, targeted antimicrobials, and metabolite-based interventions, it is particularly important to avoid equating mechanistic hypotheses directly with clinical efficacy. Genera such as Lactobacillus and Bifidobacterium contribute to the maintenance of urogenital tract homeostasis, but their benefits in PCa still lack support from large-scale randomized controlled trials. Antimicrobial therapy targeting specific microbiota is only justified when the pathogenic microorganisms, the underlying pathways, and the beneficiary population are clearly identified; otherwise, it may induce new microbial imbalances, increase the risk of antimicrobial resistance, or attenuate immune effects. The study by Uzelac et al. on the relationship between microbial dysbiosis, castration resistance, and cancer stemness, as well as the summary by Pernigoni et al. on the impact of the microbiota on treatment response in PCa, provide theoretical support for the above strategies, but these findings still require validation in longitudinal cohorts and interventional studies (87, 88).

A more mechanistically specific direction involves live biotherapeutic products and local delivery strategies. Preclinical models have shown that certain engineered bacteria, such as the patient-derived strain CP1, can target tumor sites after transurethral administration, induce immunogenic cell death, and exhibit synergistic anti-tumor effects with PD-1 blockade (89). However, drawing from broader experience in tumor microbial therapy, live biotherapeutic products still face several challenges, including strain stability, reversion to virulence, horizontal gene transfer, bacterial shedding, immunotoxicity, quality control in production, and regulatory standards (90). Therefore, future trials in the PCa field should prioritize the use of molecularly stratified populations, establish multi-layered endpoints including immune infiltration, cytokines, metabolites, and imaging/pathological responses, and adopt safety and reversibility as core evaluation criteria.

## 9 Summary and challenges

Overall, existing research has demonstrated that the urinary tract is not a sterile environment, and changes in its microbial composition are multi-dimensionally associated with the initiation and progression of PCa, remodeling of the immune microenvironment, and treatment response. PCa-associated microbial alterations have been observed in urine, prostatic fluid, semen, and prostate tissue. Certain microbial taxa and their metabolites may influence tumor biology through pathways such as chronic inflammation, TLR/NF-κB signaling, STAT3 activation, Treg/Th17 balance, macrophage polarization, DNA damage, and androgen metabolism. In addition, cancer cells can reshape the tumor microenvironment, further enriching pro-carcinogenic microbial communities. However, the current evidence remains primarily correlational and preclinical, and it is not yet sufficient to determine whether most microbial changes are a cause of PCa or a consequence of tumorigenesis. This field still requires further research to clarify the causal relationships between these factors.

Future research should first transition from case-control descriptions to longitudinal causal validation. An ideal study design would include repeated sampling cohorts before diagnosis or before and after treatment, paired sampling of urine and prostatic fluid/tissue, detailed documentation of antibiotic and lifestyle exposures, simultaneous collection of PSA, imaging data, pathological grading, treatment modalities, and recurrence outcomes. Furthermore, methods such as Mendelian randomization, mediation analysis, and external validation cohorts should be employed to distinguish between the causes and consequences of microbial dysbiosis. Second, standardized workflows suitable for low-biomass urogenital samples should be established. The sampling phase should specify the urine type, prostate massage status, source of biopsy or surgical tissue, and contamination control strategies. The experimental phase should include blank extraction controls and positive controls. The analytical phase should report quality control thresholds and model parameters. Only microbial signals that can be consistently and reproducibly observed across multiple centers and populations have the potential to serve as reliable clinical biomarkers. Third, mechanistic studies need to connect candidate microbial taxa with verifiable host responses. This can be achieved through the use of prostate organoids, primary epithelial cells, germ-free or humanized animal models, *in situ* colonization experiments, and strain knockout/complementation assays to verify whether specific microorganisms or metabolites are sufficient to induce inflammation, immune evasion, AR signaling activation, or treatment resistance. For multi-omics and AI models, emphasis should be placed on interpretability, independent test sets, and clinical decision-making benefits, avoiding the reporting of high accuracy alone without biological and clinical interpretability. Finally, clinical translation should adhere to a stratified approach: in the short term, the microbiome can be used as an exploratory auxiliary indicator in addition to PSA, mpMRI, and pathological assessment; the medium-term goal is to develop externally validated risk prediction panels and treatment response monitoring models; the long-term direction is individualized intervention based on specific microorganisms, metabolites, or engineered bacteria.
